# SOCIOECONOMIC STATUS, SOCIAL SUPPORT, AND PSYCHOLOGICAL DISTRESS IN AGING KENYANS

**DOI:** 10.1093/geroni/igad104.2011

**Published:** 2023-12-21

**Authors:** James Muruthi

**Affiliations:** College of Nursing and Health Professions, Drexel University, Philadelphia, Pennsylvania, United States

## Abstract

The incidence of psychological distress is on the rise among aging Africans. Studies have indicated that in the absence of public support, relying on informal social networks might benefit the psychological health of aging Africans. However, the associations between social support, economic status, and psychological distress remain unclear in this population. This study investigated the prevalence of psychological distress among aging Kenyans and examined its relationships with economic status (indicated by standard measures for low- and mid-income populations) and perceived social support (multidimensional and provided by family, neighbors, and religious communities). Participants from rural and urban areas of Kenya completed computer-assisted personal interviews in May 2022 (N = 376). Overall, 61% reported high psychological distress. Structural equation modeling results indicated that food insecurity (β = .236, SE = .074, p < .001), sex (β = .146, SE = .056, p < .05) and self-reported physical health (β = .386, SE = .051, p < .001) positively predicted psychological distress while educational status (β = .146, SE = .058, p < .05), and flooring material (β = .107, SE = .056, p < .05) were negative predictors. The dimensions of social support were not significantly associated with the outcome variable. The findings illuminate that psychological distress is a critical health concern for the sample and needs targeted health interventions. They also underline the critical role of economic status on the psychological distress of aging Kenyans. Future studies should further explore these relationships, especially using longitudinal and representative data.

